# Establishing standards for controlled donation after circulatory determination of death in adults: the Bucharest international European Society for Organ Transplantation consensus

**DOI:** 10.3389/ti.2026.16284

**Published:** 2026-06-04

**Authors:** Helen Opdam, Alicia Pérez-Blanco, Dale Gardiner, Richard Hasz, Nichon Esther Jansen, Matthieu Le Dorze, Alberto Sandiumenge, Giuliano Testa, Shih-Ning Then, Marinella Zanierato, Beatriz Domínguez-Gil, Gabriel C. Oniscu, Umberto Cillo, Dominique E. Martin

**Affiliations:** 1 Department of Intensive Care, Austin Hospital, Heidelberg, VIC, Australia; 2 Australian Organ and Tissue Authority, Canberra, ACT, Australia; 3 Organizacion Nacional de Trasplantes, Madrid, Spain; 4 Organ and Tissue Donation and Transplantation, NHS Blood and Transplant, Bristol, United Kingdom; 5 Gift of Life Donor Program, Philadelphia, PA, United States; 6 Department of Policy and Research, Dutch Transplant Foundation, Leiden, Netherlands; 7 Department of Anesthesia and Critical Care, AP-HP, Hôpital Lariboisière, Paris, France; 8 Université Paris Cité, INSERM U942 MASCOT, Paris, France; 9 Vall d’Hebron Research Institute, Barcelona, Spain; 10 Hospital Universitari Vall d’Hebron, Barcelona, Spain; 11 Simmons Transplant Institute, Baylor University Medical Center, Dallas, TX, United States; 12 Australian Centre for Health Law Research, Faculty of Business & Law, Queensland University of Technology, Brisbane, QLD, Australia; 13 Department of Anesthesia and Critical Care, AOU Città della Salute e della Scienza di Torino, Turin, Italy; 14 Division of Transplantation Surgery, CLINTEC, Karolinska Institutet, Stockholm, Sweden; 15 Unità di Chirurgia Epatobiliare e Trapianto Epatico, Università degli Studi di Padova, Padova, Italy; 16 School of Medicine, Deakin University, Geelong, VIC, Australia

**Keywords:** consensus, DCD (donation after circulatory death), deceased donation, donation, transplantation

## Abstract

Controlled donation after circulatory determination of death (cDCDD) is increasingly vital to expanding deceased organ donation globally, yet variability exists in clinical, legal, and ethical practices. This study utilized a Delphi consensus process involving 37 international experts to develop recommendations to guide the development and operation of adult cDCDD programs. Two survey rounds evaluated agreement on system requirements, donor identification, medical suitability, communication, end-of-life care, and ante-mortem interventions. Consensus was achieved on numerous recommendations emphasizing the need for robust legal frameworks distinct from end-of-life care decisions, multidisciplinary approaches for donor suitability assessment, and clear, sensitive communication led by trained donation professionals. Ensuring patient comfort and dignity during withdrawal of life-sustaining measures, alongside optimizing donation outcomes, was prioritized. Use of ante-mortem interventions was deemed to require careful balancing of benefits and burdens in line with patient and family preferences. The findings highlight international variability and underscore the importance of tailored protocols, education, and further research to establish an evidence base for ante-mortem interventions and improve clinical prediction of donation feasibility. These consensus recommendations aim to advance ethical, effective, and sustainable adult cDCDD programs worldwide.

## Graphical Abstract

**Figure:**
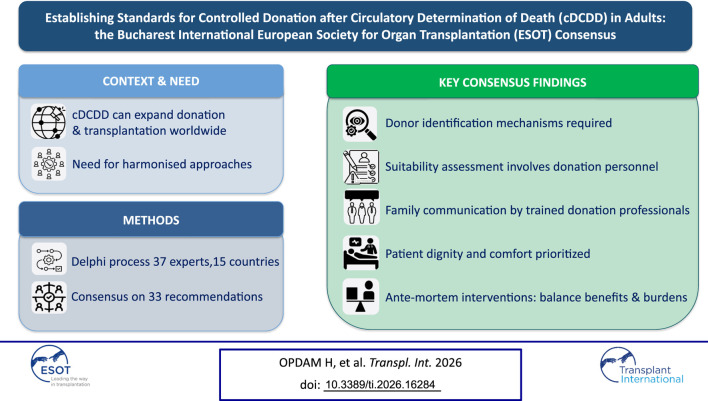

## Introduction

Controlled donation after circulatory determination of death (cDCDD) has emerged as a critical means for expanding deceased organ donation worldwide [1, 2]. Recent data reveal that cDCDD provides a significant contribution to the organ donation rates across various countries, helping to meet the rising demand for transplantation [3]. When appropriately integrated into end-of-life care planning, cDCDD broadens opportunities allowing the donation choices of patients and their families to be considered and respected. Despite its growing role, cDCDD programs face distinct ethical, clinical, and logistical challenges that set them apart from donation after neurological determination of death (DNDD). These challenges include complex decision-making processes surrounding the withdrawal of life-sustaining measures (WLSM), the process of death determination, the management of warm ischemic injury, as well as the integration of ante-mortem interventions to optimize transplant outcomes [4].

This paper presents consensus recommendations developed through a Delphi process involving international experts in adult deceased donation. The aim is to provide ethical, clinical, and operational guidance for establishing and sustaining adult cDCDD programs, considering the variability in legal frameworks, healthcare infrastructure, and cultural contexts. Emphasis is placed on maintaining integrity of end-of-life care, ensuring informed and sensitive communication with patients and families, optimizing donor identification and assessment, and balancing patient-centered care with the imperative to maximize donation opportunities. This work seeks to support national programs in delivering effective, ethical, and sustainable cDCDD pathways within the broader deceased donation landscape.

## Materials and methods

The work described in this paper formed part of the European Society for Organ Transplantation (ESOT) Donation after Circulatory Determination of Death (DCDD) Consensus Project, a multi-phase initiative designed to establish standardized terminology and recommendations in controlled DCDD practice. The Delphi process applied in the overarching DCDD Consensus project is outlined in detail elsewhere [5]. This paper is the result of one of four subcommittees, with this group tasked with developing consensus on best practice standards and recommendations for adult DCDD.

A steering committee comprised of 9 members with expertise in adult deceased donation developed a questionnaire addressing a range of topics identified as priorities via literature review and group discussions. The committee identified expert panellists according to the criteria described in Martin et al. [5], who were invited to participate in the Delphi process. The two coordinators were excluded from the expert panel.

Two survey waves were conducted using an online questionnaire administered by the independent company Adelphi Targis. All panellists who completed the first questionnaire were invited to complete the second wave survey. The questionnaire was refined for the second wave as previously described [5].

In each survey round panellists indicated their level of agreement with a series of statements using a Likert scale (1-9, strongly disagree–strongly agree); responses were analysed using descriptive statistics with “disagreement” assigned to ratings 1–3, “neither agree nor disagree” 4-6, and “agreement” to ratings 7–9. Participants could alternatively indicate if a question was not relevant to their expertise. Responses from those who indicated a lack of relevant expertise were removed from the denominator when evaluating consensus on specific questions. Statements that reached 75% agreement were deemed to achieve consensus. The results from the second round relating to definition of terms are reported elsewhere [5].

## Results

Thirty-seven experts from 15 countries completed the first round of the Delphi process of whom 35 completed the second. Panel demographics are shown in Table 1.

**TABLE 1:** Expert panel demographics.

Panelist characteristics	First-wave	Second-wave
​	n = 37	n = 35
Age		
18–30 years	0.0%	0.0%
31–40 years	10.8%	11.4%
41–50 years	24.3%	22.9%
51–60 years	35.1%	37.1%
>60 years	29.7%	28.6%
Gender		
Male	64.9%	62.9%
Female	35.1%	37.1%
Country of employment		
Argentina	5.4%	5.7%
Australia	10.8%	11.4%
Canada	5.4%	5.7%
China	5.4%	2.9%
France	8.1%	8.6%
Italy	10.8%	11.4%
Japan	2.7%	0.0%
Netherlands	8.1%	8.6%
New Zealand	2.7%	2.9%
South Africa	2.7%	2.9%
Spain	8.1%	8.6%
Sweden	2.7%	2.9%
United Kingdom	10.8%	11.4%
United States	13.5%	14.3%
Uruguay	2.7%	2.9%
Primary specialty		
Intensivist/critical care physician	40.0%	39.6%
Transplant or donation coordinator	18.0%	18.8%
Transplant surgeon	14.0%	12.5%
Anaesthesiologist	6.0%	6.3%
Ethicist or legal scholar	6.0%	6.3%
Others*	16.0%	16.7%
Primary patient population		
Adult medicine	67.6%	65.7%
Both paediatric and adult medicine	16.2%	17.1%
Not applicable to my role	16.2%	17.1%
Paediatrics	0.0%	0.0%
Primary sector of clinical practice for previous 12 months		
Public healthcare	73.0%	71.4%
Private healthcare	0.0%	0.0%
Both	16.2%	17.1%
Not Applicable	10.8%	11.4%
Type of hospital in which work		
Academic hospital	59.5%	60.0%
Non-academic hospital	8.1%	5.7%
Other academic centres and institutions	16.2%	17.1%
Other	16.2%	17.1%
Duration of experience with deceased donation or transplantation		
≥10 years	86.5%	85.7%
5–9 years	10.8%	11.4%
<5 years	2.7%	2.9%
Author contributions to academic publications in deceased organ donation or transplantation		
Yes	94.6%	94.3%
No	2.7%	2.9%
Not applicable	2.7%	2.9%
Participation as a principal investigator in a clinical trial relating to deceased organ donation or transplantation		
No	59.5%	62.9%
Yes	37.8%	34.3%
Not applicable to my role	2.7%	2.9%

* Values may not add up to 100% due to rounding errors.

Consensus was achieved on 120 statements in the first round and on an additional 8 statements in the second round. The 39 recommendations summarized in Tables 2–7 reflect those statements for which consensus was reached, with some statements combined for efficiency.

**TABLE 2:** Consensus recommendations regarding system requirements for adult cDCDD programs.

1. When evaluating the potential costs and benefits of introducing or expanding cDCDD programs, careful attention should be given to the local context and the potential impact of legislation, available technologies, and cultural norms on opportunities for cDCDD and utilisation of organs
a. cDCDD programs should be introduced in a manner that prioritises, where possible, opportunities for donation following neurological determination of death (DNDD)
b. Careful introduction of cDCDD programs may result in additional DNDD, as a proportion of patients who are evaluated as potential donors via cDCDD will proceed to DNDD
2. A legal framework allowing for withdrawal of life-sustaining measures (WLSM) and ethical guidance supporting decision-making about WLSM as part of routine end-of-life care should be established independently of efforts to develop cDCDD programs
3. Legislation for deceased donation should not detail specific permissible ante-mortem interventions, so as to avoid unintended constraints on future practice given the continuous evolution of medicine and transplantation
4. Potential conflicts between components of laws governing medical treatment decision-making, deceased donation, and research that may be applicable in the context of end-of-life care and cDCDD should be resolved
5. Public education about deceased donation should include specific education and information pertaining to the cDCDD pathway, to promote public understanding and awareness of how this may differ from other donation pathways
6. Requirements for successful implementation of cDCDD programs include
a. A legal framework
a. Permitting the determination of death by circulatory criteria
b. Establishing mechanism(s) for consent or authorisation of ante-mortem interventions for donation
c. That enables research relating to cDCDD, e.g., by providing mechanisms for consent on behalf of persons who lack decision-making capacity
b. Education for clinicians regarding clinical, ethical, and legal aspects of cDCDD.
c. Inclusion of cDCDD within the portfolio of governmental health authorities
d. Ethical guidance to support health professionals involved in decision-making about end-of-life care in the context of cDCDD.

**TABLE 3:** Consensus recommendations for preserving and identifying opportunities for cDCDD in adults.

1. Supportive treatments should be maintained in patients potentially suitable for cDCDD until donation can be discussed with the patient or their SDM, unless medical suitability for cDCDD is already excluded
2. Clinical management should be viewed as a continuum of care that includes family support throughout the donation process and appropriate comfort measures for patients who are potential cDCDD donors
3. Identification of potential cDCDD donors should be a recognized responsibility of
a. Any healthcare professional caring for patients in a critical condition who are receiving life sustaining measures, and b. Intensive care and emergency medicine teams
4. Clinical triggers (tailored to local practices) should be developed to ensure timely
a. Identification of all potential cDCDD donors, including patients in whom death is considered inevitable following planned WLSM, and routine communication of these cases to the relevant donation personnel, to assess their suitability for cDCDD.
b. Consideration of organ donation options as part of decision-making about end-of-life care
5. Adult cDCDD protocols require guidance that addresses
a. Criteria identifying possible donors whose medical suitability to donate should be evaluated
b. Criteria defining medical suitability for donation
c. When and to whom information about possible donors should be communicated for assessment of medical suitability for donation
d. When and by whom patients and/or their substitute decision maker (SDM) should be contacted to discuss the possibility of cDCDD.
e. Care of the patient while initial assessments of suitability for donation and/or donation decision-making are made

**TABLE 4:** Consensus recommendations for assessment of medical suitability for cDCDD in adults.

1. Guidance for the assessment of medical suitability for cDCDD in individual cases should be established at the national level and should be informed by understanding of relevant factors that may vary between healthcare institutions
2. When evaluating medical suitability for cDCDD, individual characteristics of the potential donor as well as local healthcare system factors that may influence the probability of successfully recovering and utilising organ(s) in transplantation should be assessed
3. Criteria used in the assessment of individual suitability for cDCDD should distinguish between potentially modifiable healthcare system factors that may influence the feasibility of cDCDD and the clinical characteristics of individual patients that may influence the probability of death occurring within a specified time following WSLM.
4. When evaluating medical suitability for cDCDD
a. Assessment of suitability to donate specific organs is recommended
b. Decisions about suitability should not rely on a single method of prediction of time to death after WLSM because the clinical assessment of an attending intensivist or scoring systems (e.g., University of Wisconsin and the UNOS) is imprecise and often unreliable
c. Individual evaluation should be multi-disciplinary and should involve the treating clinician, donation personnel and, if required, consultation with transplant teams
5. Where lawful, it is ethically acceptable for donation personnel to make a preliminary assessment of medical suitability for cDCDD using information obtained from the treating team, without consulting the patient or their SDM, to determine whether donation should be offered
a. If additional information about the potential donor or additional investigations are needed to assess their medical suitability for cDCDD, beyond what is available as part of the patient’s medical care, the patient and/or their SDM should be approached to discuss the possibility of cDCDD before further assessment of medical suitability
6. Where there are logistic or other system constraints that mean it is not feasible to attempt cDCDD, data should be collected and used to inform efforts to overcome barriers to cDCDD in future

**TABLE 5:** Consensus recommendations for communication about donation relating to cDCDD in adults.

1. Where clinically relevant, information about potential opportunities for cDCDD should routinely be provided to patients, their families, and/or SDMs as part of end-of-life decision-making
2. Unless discussion of donation is raised independently by the patient, their family or SDM
a. The possibility of cDCDD should not be raised with them until it is clear they understand that death will soon occur
b. Discussions about cDCDD should be initiated where possible by a trained donation professional who is not involved in the clinical care of the potential donor
3. Donation discussions should be led where possible by a trained donation professional who is not involved in the clinical care of the potential donor
4. It is the responsibility of the treating physician caring for a critically ill patient to communicate with the patient’s family and/or SDM about decisions regarding WLSM at the end-of-life
5. Communication with patients (where possible and appropriate), their families and/or SDM should ensure that decisions about WLSM are not influenced by the possibility of organ donation
a. Information about cDCDD should only be provided to patients, their family and/or SDMs after a decision has been made about WLSM.
b. Formal decisions about cDCDD resulting in lawful consent for donation should only be made after a decision has been made regarding WLSM.
6. The disclosure of information about cDCDD protocols to patients, and/or their SDM during decision-making about donation should satisfy the requirements for lawful consent in the relevant jurisdiction
a. The scope, depth, and detail of information about cDCDD protocols disclosed during decision-making about donation should be tailored to the preferences of the patient and/or their SDM
b. Detailed information about the process of organ removal or invasive post-mortem interventions such as NRP should only be provided to patients, their family and/or SDM by a trained donation specialist
7. When a patient or SDM is making a decision about cDCDD, they should routinely be provided with some information about
a. Options for donated organs or tissues to be used in research if not utilised in transplantation
b. The possibility of “stand down”, when an attempt at DCDD is aborted because death has not occurred within a timeframe that enables organ eligibility for transplantation, as defined by local protocols
c. Potential ante-mortem and post-mortem interventions that may be used to increase the probability of successful donation for transplantation
d. The potential implications of end-of-life care choices for cDCDD, i.e., how some choices may influence the probability of successful recovery and utilisation of organs for transplantation (see recommendation 8 below)
8. If the patient’s known or estimated preferences regarding end-of-life care (e.g., timing or location of WLSM) may negatively impact opportunities for donation or utilisation of organs
a. They or their SDM should be informed, if relevant, that it may not be feasible to support donation
b. It is important they or their SDM understand that fewer organs may be able to be donated and successfully transplanted
9. When the availability of surgical teams influences the optimal time for WLSM, the options and likely impact on donation and transplantation outcomes should be explained to the patient or their SDM who will make an informed choice regarding the timing of WLSM.
10. When options are available for the location of WLSM that influence the likely success of donation of organs for transplantation, the patient or their SDM should make an informed choice regarding the location
11. In patients with a devastating brain injury who do not meet criteria for neurological determination of death (NDD) at the time of donor referral, discussions about cDCDD should include information about the possibility, where relevant, of donating via the NDD pathway
12. Guidance should be developed that establishes how decisions should be made about donation and end-of-life care by SDMs in accordance with relevant laws, e.g., making clear when the person’s known or estimated preferences should take priority in decision-making
a. When it is lawful to do so, a SDM should make decisions about end-of-life care and/or cDCDD based on what they believe the patient would decide if they were able to make their own decision
b. When making decisions about potential aspects of end-of-life care that may influence the likelihood of successful donation of organs following cDCDD, the known or estimated preferences of the patient should determine choices where possible
c. If a SDM believes that what is best for the patient conflicts with what the patient would decide if they were able to make their own decision about cDCDD, the SDM should make the decision they believe the patient would make, provided this approach is lawful

**TABLE 6:** Consensus recommendations for managing the WLSM and end-of-life care process in cDCDD in adults.

1. The clinical approach to end-of-life care prior to and during WLSM should be consistent with that of routine end-of-life care in the absence of cDCDD including provision of comfort care (e.g., sedatives and analgesia)
a. The WLSM process that includes cessation of cardio-respiratory supports and provision of comfort care (e.g., sedatives and analgesia) should be undertaken by the treating clinical team and not staff overseeing or involved in the donation process
b. Comfort care (e.g., sedatives and analgesia) should be provided as per routine care in the absence of cDCDD.
c. Where required, analgesia and/or sedation should be provided for ante-mortem interventions for donation as they would be for the same interventions if performed for therapeutic purposes
d. Family members should always be offered the option of being present during WLSM and until death is determined
2. Staff overseeing or involved in cDCDD for an individual may provide information or advice to the patient’s treating team regarding how end-of-life care choices (e.g., comfort care provision, mode of WSLM) may impact the likelihood of successful donation of organs for transplantation
3. The clinical approach to end-of-life care prior to and during WLSM should optimise the likelihood of successful donation of organs for transplantation except when this may negatively impact the comfort of the patient
a. The location of WLSM should optimise quality of end-of-life care for the patient and their family and/or SDM, as well as the success in the recovery and utilisation of donated organs
b. Where possible, the timing of WLSM should allow for optimal completion of the necessary donor assessment and preparation for the donation surgery
4. Prior to WLSM in the context of cDCDD, staff responsible for overseeing cDCDD should meet to confirm or reconfirm
a. Introductions are made for all personnel involved
b. Roles and responsibilities of each staff member
c. Donor paperwork and legal authorisations have been reviewed
d. Planned organ retrieval and surgical steps including subsequent tissue retrieval
e. Agreement on procedures if death does not occur in the timeframe
f. Timing of WLSM
g. Finalisation of operating theatre requirements

**TABLE 7:** Consensus recommendations regarding use of ante-mortem interventions for cDCDD in adults.

1. Where available, lawful, and clinically relevant, use of ante-mortem interventions should be considered if their potential benefits outweigh their potential burdens or risks in a person for whom donation is known or estimated to be a goal
2. When evaluating the potential benefits of using an ante-mortem intervention, the potential benefits of not using the intervention, such as achievement of a person’s wish to avoid clinical procedures at the end-of-life should also be considered
When evaluating the potential harms of using an ante-mortem intervention, the potential harms of not using the intervention, such as reduced likelihood of achieving a person’s donation goals should also be considered
The potential benefits of using ante-mortem interventions should be considered inclusive of their potential impact on transplant recipients, on fulfilment of a person’s known or estimated goal to donate organs for transplantation, and on the person’s family
3. Ante-mortem interventions should only be considered where there is a clear clinical justification for their potential to optimise donation and transplant outcomes
a. Research should be undertaken to provide an evidence base for the potential benefits and harms of ante-mortem interventions
b. Where lawful, ante-mortem interventions of uncertain benefit may be considered if they are minimally burdensome and pose no substantive risk of harm to the patient
4. Even if specific consent for an ante-mortem intervention is not required, it is best practice to provide some information about the intervention to the patient, their family or SDM as part of routine communication about the donation process
5. Information about the following aspects of ante-mortem interventions is relevant for health professionals and patients or their SDMs when making decisions about use of potential interventions
a. The potential to cause the patient or family discomfort or harm
b. The invasiveness of the intervention, meaning the level of physical intrusion in the body
c. Whether the intervention is commonly used in critically ill patients in the ICU.
d. The extent to which the intervention may alter the usual end-of-life care process for the patient
e. The likely impact of the intervention on the probability of successful donation and transplantation of organs
6. The following information is important in guiding decision-making about use of ante-mortem interventions in cDCDD where these are lawful and clinically indicated
a. The known or estimated importance of achieving successful donation and transplantation to the patient
b. The importance of the patient’s known or estimated end-of-life goals that may be impacted by use of specific ante-mortem interventions
c. The importance of achieving successful donation and transplantation to the family of the patient
d. The importance of the patient’s family’s goals regarding the end-of-life experience that may be impacted by use of specific ante-mortem interventions

## Discussion

The broad agreement on most issues likely reflects the extent to which cDCDD practices have already been carefully examined [4]. This discussion provides a wider context, addressing the systemic and programmatic requirements for adult DCDD. It concentrates on the key elements of the cDCDD process.

### System requirements for adult cDCDD programs

In 2024, the Global Observatory on Donation and Transplantation (GODT) reported 47,180 deceased organ donors from 78 of the 92 contributing countries [6]. Among these, 26 countries reported DCDD activity accounting for 13,366 donors, or 28% of the global total. Most DCDD cases were Maastricht type III (94%), where organ donation follows the WLSM [7, 8]. DCDD contribution to deceased donor activity within countries was highly variable: 66% in the Netherlands, 52% in Switzerland, 51% in Spain, 50% in Belgium and the UK, 43% in the United States, 36% in Australia and Canada, 31% in China, 25% in Sweden, 24% in Denmark, 23% in New Zealand, 21% in Ireland, and 20% or lower in Finland, Austria, Italy, France, Portugal, Czech Republic and Norway. DCDD is thus recognised as a key area for expansion of deceased donation activity [1, 2].

Establishing cDCDD programs requires comprehensive legal and regulatory frameworks (see Table 2). It is essential that laws and associated practices in end-of-life care allow the discontinuation of life-sustaining measures, such as mechanical ventilation and cardio-respiratory support, when such treatments are deemed no longer beneficial or excessively burdensome. This applies in situations where death is inevitable or when, in alignment with the patient’s wishes and family agreement, meaningful recovery is no longer possible. Importantly, such clinical practices should be grounded in the patient’s best interests, which include consideration of their preferences, and implemented independently from any consideration of potential suitability for donation, thus maintaining the integrity of end-of-life care decisions.

There was consensus that cDCDD programs should be introduced in a manner that prioritises opportunities for DNDD and that implementing a cDCDD program can augment DNDD. In some countries, an increase in rates of DCDD has coincided with a decline in DNDD, prompting concerns about premature diversion of potential DNDD candidates to the DCDD pathway, perhaps before allowing sufficient time for brain death to occur in patients with severe brain injury. GODT data from fifteen countries illustrate two main trends: in nine countries (such as Australia, Canada, Italy, and Sweden) DNDD rates have remained stable or increased after DCDD implementation, while in six (including Spain, Belgium, the Netherlands, and France) DNDD rates fell as DCDD rose [9]. Traditional DCDD practices with standard rapid recovery and cold storage generally yield fewer transplantable organs per donor and can result in inferior transplant outcomes for organs like the liver compared with DNDD [10]. Innovations such as use of perfusion technologies have the potential to reduce such disparities [11, 12]. Increasing DCDD activity may also be the result of changing end-of-life care practices independent of organ donation considerations, such as a shift to earlier discussions with families about prognosis and subsequent WLSM [13–15]. However, a notable proportion of patients considered for cDCDD ultimately proceed to the neurological determination of death (NDD), thus becoming DNDD donors. Some of these individuals might have been excluded from donation in the absence of a cDCDD pathway [16].

cDCDD is only feasible through the provision of ongoing supportive treatments and interventions prior to death that are performed solely for the purpose of donation, (see the consensus definition of ante-mortem interventions outlined below). The use of ante-mortem interventions in DCDD programs may require clear legislation as well as comprehensive ethical and practical guidance and their absence can act as a significant barrier to DCDD [17]. Where the lack of clear laws or guidelines has proven a barrier, legislation and guidelines should be introduced to explicitly address the permissibility of ante-mortem procedures *per se* and set out clear requirements for consent or authorisation of their use, which may involve substitute decision makers (SDMs). However, legislation should not specify permissible ante-mortem interventions to avoid unintended constraints on future practice given the continuous evolution of medical practice.

Consensus highlighted the importance of robust ethical guidance to support health professionals involved in end-of-life decisions or caring for donors within the context of cDCDD. Such guidance must ensure the clear separation of decisions regarding the cessation of non-beneficial treatments including WLSM, and those concerning organ donation, to prevent conflicts of interest [2, 18]. The panel also agreed on the critical need for comprehensive education for clinicians on cDCDD.

### Preserving and identifying opportunities for cDCDD in adults

There was strong consensus that healthcare professionals should routinely consider organ donation as an integral part of end-of-life care, with donor identification recognized as a shared responsibility across clinical teams (see Table 3). There was support for clinical triggers or other mechanisms to aid the routine and timely communication with donation personnel for all patients potentially suitable for cDCDD, with supportive treatments maintained until donation could be discussed with the patient or their SDM, or cDCDD excluded due to lack of medical suitability.

Determining suitability for cDCDD can be more difficult than for DNDD candidates. Patients suitable for cDCDD include those with severe brain injuries who do not satisfy criteria for NDD, and patients with other irreversible disease processes including cardiac, respiratory and liver failure, and neuromuscular conditions (e.g., amyotrophic lateral sclerosis, high spinal cord injury). Clinicians more familiar with DNDD may overlook potential donors in these other clinical scenarios. Furthermore, judgements about the likelihood of death occurring after WLSM in a timeframe that allows for donation are difficult. Therefore, exploring cDCDD opportunities requires the acceptance that a proportion of attempted DCDDs may not proceed for this reason (see below) [19, 20].

Assessing the suitability of specific organs for transplantation also requires current knowledge of acceptance criteria and may necessitate advice from transplant units. Suitability can vary depending on whether there are recipient candidates in urgent need of transplantation, for whom the use of expanded criteria donor organs that may not be otherwise considered could be life-saving. Determination of medical suitability consequently requires the expertise of donation and transplantation personnel, and treating clinicians should not unilaterally exclude patients as unsuitable for donation.

Clinical triggers or similar mechanisms for initiating a referral to donation personnel can be helpful in ensuring the timely identification of all potential donors [21, 22]. For cDCDD, these need to be sufficiently broad to capture all potential donor opportunities. They are best tailored to local circumstances, considering donor suitability criteria that are contingent on local, regional or national transplant units organ acceptance practices, the point in the patient hospital course at which communication with donation personnel should occur, and the best mechanism for communication with donation personnel. In countries such as Spain, where cDCDD may be facilitated in patients approaching death outside critical care settings through the initiation of intensive care measures (intensive care to facilitate organ donation, ICOD), broader identification criteria are required to capture this donor pool [23, 24].

The communication with donation personnel should take place prior to any reduction in supportive treatments, which might otherwise compromise organ function and limit subsequent opportunities for donation. This should also be before any agreed time with the family for WLSM, which would leave insufficient opportunity to determine suitability, approach family to offer donation, and arrange the logistics of donation.

Overall, there was strong consensus that healthcare professionals caring for critically ill patients must take active responsibility for donor identification, supported by routine consultative mechanisms with donation experts. However, medical suitability should be determined by donation personnel, in consultation with transplant units where necessary, rather than by treating healthcare professionals. This promotes normalization of organ donation as a standard component of end-of-life care decision-making, enhancing donation rates while maintaining ethical and clinical integrity.

### Assessment of medical suitability for cDCDD in adults

Suitability for cDCDD varies widely across systems and depends on several factors, including medical eligibility, end-of-life care practices, and the use of organ preservation techniques (see Table 4). Medical suitability for donation, whether DNDD or DCDD, includes an assessment of disease transmission risks and the function of individual organs, with differing eligibility between countries and even local transplant units depending on risk tolerance and the maturity of the transplantation programs. For cDCDD, an additional element is whether death will occur soon enough after WLSM to permit donation, and options for mitigating the impact of warm ischaemic injury through machine perfusion technologies [25].

Judging the likelihood of death occurrence soon after WLSM is imprecise, as this is influenced by many patient characteristics (see Box 1). Scoring systems incorporating these elements have been developed to support decision making, however these may not be better than the judgement of an experienced attending intensivist [19, 20, 26–28]. Given the imprecision and the unreliability of such assessments, the respondents agreed that suitability for donation should not rely on a single method of predicting the time to death post WLSM.

Box 1 **Factors influencing opportunities for adult cDCDD.**

1. Clinical characteristics of the potential donor that may influence the duration of time between WLSM and circulatory arrest, and hence functional warm ischaemia time (FWIT), include:
  - Presence and severity of a devastating brain injury, including depth of coma and number of absent brainstem reflexes.
  - Airway patency (e.g., airway pathology, sleep apnoea, obesity).
  - Dependence on mechanical ventilation and oxygen requirements prior to WLSM.
  - Dependence on vasoactive and/or mechanical circulatory supports.
2. Several local healthcare system factors and choices made by patients or SDMs regarding end-of-life care may influence the likely duration of acceptable FWIT and cold ischaemia times in the setting of cDCDD. These include:
  - End-of-life care practices, including the mode of WLSM (e.g., extubation), the approach to provision of comfort care with intravenous sedatives or analgesic agents (e.g., use of deep sedation versus the minimal amount for symptom relief).
  - Location of WLSM and consequent time required to move the deceased person to the operating room.
  - Availability and use of machine perfusion technologies, including *ex situ* organ perfusion and *in situ* normothermic regional perfusion.
  - Availability of surgical expertise and operating theatre access.
  - Travel distances and transport options for recovery teams, donation staff, and recovered organs.
3. Additional factors that may influence decisions to attempt cDCDD in specific cases include:
  - Characteristics of the potential donor that may influence the utilisation or likely outcomes of transplantation if an organ(s) is recovered, e.g., advanced age or non-standard risk for infectious disease transmission.
  - Relevant transplant unit acceptance practices (e.g., ability to transplant extended criteria donor organs into suitable candidates, noting practices will vary for different organs).
  - Characteristics of potential organ recipients, e.g., urgency and severity of need for transplantation, likelihood of receiving an organ from another donor.
  - Level of SDM, family, or patient support for donation.

The approach to sedation and analgesia at the time of WLSM can also affect the time to death. Sedation may be administered either with the primary aim of symptom relief or as continuous deep sedation until death, and this distinction, combined with national stand down policies, has an important impact on donation outcomes. Stand down time refers to the timepoint after WLSM when donation efforts are abandoned if death has not occurred and organs are no longer considered eligible for transplantation [5]. In France and Spain, where continuous deep sedation until death is routine practice, death within stand down periods of 90–180 min is almost universal [29–31]. By contrast, in the UK and Australia, where medications are adjusted to manage symptoms instead of inducing deep unconsciousness, 20% of attempted cDCDD cases in the UK and 30% in Australia do not reach circulatory death within the required stand down times of 3 h and 90 min, respectively, resulting in no donation [32, 33]. Longer stand down times may allow a higher proportion of attempted cases to result in donation, with satisfactory transplant outcomes supported by the use of *in situ* normothermic regional perfusion (NRP) and *ex situ* machine perfusion devices.

The efficiency of cDCDD case selection is directly influenced by how strictly potential donors are chosen; limiting attempts to those highly likely to die rapidly after WLSM improves conversion rates but may reduce overall donation opportunities, while broader inclusion criteria enlarge the donor pool but leads to a lower proportion of actual donors per number of attempted DCDD cases.

There was agreement that guidance for the assessment of medical suitability for cDCDD in individual cases should be established at the national level, informed by an understanding of relevant factors that may differ across healthcare institutions. In evaluating medical suitability, both the individual characteristics of the potential donor and local healthcare system factors influencing the likelihood of successful organ recovery and transplantation must be considered (see Box 1). Healthcare system factors included end-of-life care practices, availability of perfusion technologies, and human resource expertise and availability, as well as logistical factors such as travel requirements. Notably, another factor that fell just below the consensus threshold with 74.3% agreement, was the availability and use of various ante-mortem interventions.

The criteria for individual donor assessment should clearly distinguish between potentially modifiable system-related factors affecting the feasibility of cDCDD and the clinical characteristics of the patient that determine suitability. Where there are system constraints limiting the ability to proceed with cDCDD, it was agreed that capturing data from such events could inform efforts to overcome barriers and improve future opportunities for cDCDD.

### Communication about donation relating to cDCDD in adults

Decisions regarding cDCDD should be integrated thoughtfully into end-of-life care, ensuring ethical and legal standards are upheld throughout the process. There was consensus that, when medically relevant, information about cDCDD opportunities should be routinely offered to patients (where possible and appropriate), families, and SDMs as part of comprehensive end-of-life discussions (see Table 5).

A broadly accepted principle in all forms of deceased donation is that, before the possibility of donation is introduced, families should understand that death has occurred (NDD) or will occur, either as a clinical inevitability or as a consequence of decisions about end-of-life care. This principle is especially important in cDCDD, where donation decision-making always precedes death and must be separated from decision-making about WLSM.

There was agreement that it remains the treating physician’s responsibility to communicate with the patient’s family or SDM about decisions regarding WLSM. To preserve the integrity of end-of-life decisions, discussions with the patient’s family or SDM about organ donation should follow and not precede the decision about WLSM, and lawful consent formalizing donation should only be sought after this point.

There was consensus that discussions about donation should be initiated and led where possible by a trained donation professional who is not involved in the clinical care of the potential donor. The importance of clinicians with relevant expertise supporting informed decision-making about donation is well established [34–36]. Research favours the involvement of donation professionals due to higher family satisfaction with communication about donation and also consent rates [23, 37, 38]. A collaborative approach, where both the treating clinician and donation personnel together communicate with families about donation is an effective model [39–41]. There was high agreement that detailed, technical explanations about organ removal, post-mortem interventions, or specific protocols like NRP should only be conveyed by donation professionals.

A key rationale for staff other than the treating clinician raising donation is to avoid a perceived conflict of interest, if the same clinician is part of decision-making about end-of-life care and donation [42]. However, once the decision for WLSM has been made, concerns about conflicts may diminish, and trusted treating clinicians could be well positioned to raise the topic of donation due to their established rapport with the family [42].

There was consensus that patients (where possible and appropriate), or SDMs considering cDCDD should also receive information about options for organs or tissues to be used in research, the possibility of ‘stand down’ if death does not occur in time for donation, and interventions (ante- or post-mortem) that may enhance the likelihood of successful donation. It was agreed that the potential impact of certain end-of-life care choices, including timing and location of WLSM, on organ recovery and transplantation success needs to be clearly explained.

If a patient’s end-of-life care preferences may compromise donation opportunities or limit transplantable organs, this should be communicated sensitively to the SDM (or the patient if conscious), highlighting potential impacts on donation feasibility and outcomes. When the timing or location of WLSM is affected by the availability of surgical teams or donation logistics, options and implications should be discussed openly to facilitate informed choices.

For patients with devastating brain injuries who do not meet criteria for the NDD at the time of donor referral, cDCDD discussions should highlight, the possibility of donation through NDD pathways, if the patient subsequently meets NDD criteria.

Nationally developed guidance should clarify how SDMs should make decisions, ensuring that actions align with the patient’s expressed or presumed wishes whenever lawful. SDMs should prioritize the known or estimated preferences of the patient over their own judgement, especially regarding aspects of end-of-life care that could influence donation outcomes, as long as it is legally permissible [24, 43].

### Managing the WLSM and end-of-life care process in cDCDD in adults

There was consensus that the approach to end-of-life care during and prior to WLSM in the context of cDCDD should align with established standards of routine end-of-life care. This includes the provision of sedation and analgesia to ensure patient comfort and dignity (see Table 6). Importantly, the WLSM and delivery of comfort care should be conducted exclusively by the treating clinical team and not personnel directly involved in the organ donation process. Comfort care for donation-related interventions before death should be equivalent to that provided when similar interventions are undertaken for therapeutic reasons. Families should be offered the option to be present during WLSM and until death is determined, supporting both transparency and emotional needs.

It was acknowledged that staff involved in cDCDD can advise the treating clinical team on how end-of-life care decisions may impact the success of organ donation, although the treating team retains primary responsibility for clinical care. This ensures that patient-centered care and comfort remain paramount, even as considerations about donation are factored in. The clinical approach should aim to enhance donation outcomes, without compromising patient comfort. For instance, WLSM timing and location decisions should balance patient and family needs with logistical requirements for organ recovery.

High support was expressed for all staff to meet before initiating WLSM to confirm roles, review donor documentation and legal authorizations, and ensure consensus on organ and tissue retrieval procedures. Teams should also establish contingency plans if death does not occur within the expected timeframe for donation. This preparation ensures a smooth, respectful process for patients and families while maximizing the efficiency and outcomes of cDCDD.

Overall, consensus supported practices that maintain the primacy of patient comfort and dignity during end-of-life care, while thoughtfully coordinating with donation teams to optimize transplantation outcomes.

### Use of ante-mortem interventions for cDCDD in adults

It was agreed that “ante-mortem intervention for donation” should be defined as “any activity, procedure, or investigation undertaken before death for the purpose of organ and tissue donation that would not otherwise occur, including those to determine, maintain or improve the viability of organs for transplantation.” cDCDD by necessity requires some ante-mortem interventions, such as continuing cardio-respiratory supports until the logistics of donation surgery can be organised, and blood sampling for infectious disease screening and tissue typing for matching to recipient candidates.

Respondents concurred that there are several aspects of ante-mortem interventions to consider in decision-making about their use, including the potential to cause discomfort or harm, the invasiveness of the intervention, whether the intervention is commonly used in ICU, whether its use alters the end-of-life care of the patient, and its importance for the success of donation and transplantation. Acceptability may also be influenced by the strength of support for donation from the potential donor and their family (see Table 7).

There was also support for disclosure of some information about ante-mortem interventions with the patient (if applicable), their family or SDM as part of routine communication about the donation process, even if specific consent for an ante-mortem intervention is not required. Although short of the 75% consensus threshold, 74.3% of panellists agreed that, “Unless required by law, when a decision has been made to proceed with donation, consent may be presumed for ante-mortem interventions that do not involve additional, physically invasive clinical procedures and which are necessary conditions of any deceased donation, such as tissue typing tests for organ matching.” A potential implication is that specific consent should be obtained for invasive clinical procedures that are not part of the standard deceased donation process. This is supported by prior reviews of clinician attitudes to ante-mortem interventions [44, 45] and consistent with some national approaches, for example, the UK Donor Actions Framework for peri-mortem interventions related to deceased organ donation and ICOD in Spain that may involve intubation and the initiation of other intensive care measures [23, 24, 46].

A central focus of the consensus discussions was the broader purpose of ante-mortem interventions; not only their commonly emphasised potential benefits for transplant recipients but also their potential to serve the interests of the patient-potential donor and their family by supporting their donation goal(s) [44, 47]. There was consensus that “The potential benefits of using ante-mortem interventions should be considered inclusive of their potential impact on transplant recipients, on fulfilment of a person’s known or estimated goal to donate organs for transplantation, and on the person’s family.” Importantly, participants in the second round agreed that research should be undertaken to provide an evidence base for the potential benefits and harms of ante-mortem interventions. They expressed the desire for robust evidence to support informed decision-making, allowing donation authorities to adequately communicate with families about the potential trade-offs involved.

Strengths of this report include a robust methodology, with recommendations developed through international expert consensus; a key limitation is that they are based largely on expert opinion. Future work to evaluate the practical usefulness of these recommendations could include administering the survey to healthcare professionals considering the initiation of a cDCDD program, to identify which recommendations are most relevant and those that may be controversial or challenging in practice.

## Conclusion

The advancement of cDCDD programs could significantly enhance the availability of organs for transplantation globally, yet it requires careful consideration of clinical, ethical, and logistical factors. The consensus guidance presented here emphasizes the primacy of patient comfort and dignity throughout end-of-life care, the importance of clear and compassionate communication with families, and the need for multidisciplinary collaboration across clinical and donation teams. National frameworks and tailored protocols are indispensable for addressing variability in healthcare settings and ensuring lawful, ethical practice.

Future efforts should prioritize building an evidence base around ante-mortem interventions, refining predictive tools for the time to death after WLSM, and facilitating education to improve clinical and donation team competency. There may be a need for professional bodies, healthcare organisations and governments to advocate for legal frameworks to be updated to facilitate implementation of consensus guidance. By harmonizing best practices and addressing systemic challenges, cDCDD programs can be effectively integrated into transplantation systems worldwide, ultimately improving organ availability while respecting the rights and values of donors and their families.

## Data Availability

The raw data supporting the conclusions of this article will be made available by the authors, without undue reservation.
