# Correction: Establishing standards for controlled donation after circulatory determination of death in adults: the Bucharest international European Society for Organ Transplantation consensus

**DOI:** 10.3389/ti.2026.17395

**Published:** 2026-07-29

**Authors:** Helen Opdam, Alicia Pérez-Blanco, Dale Gardiner, Richard Hasz, Nichon Esther Jansen, Matthieu Le Dorze, Alberto Sandiumenge, Giuliano Testa, Shih-Ning Then, Marinella Zanierato, Beatriz Domínguez-Gil, Gabriel C. Oniscu, Umberto Cillo, Dominique E. Martin

**Affiliations:** 1 Department of Intensive Care, Austin Hospital, Heidelberg, VIC, Australia; 2 Australian Organ and Tissue Authority, Canberra, ACT, Australia; 3 Organizacion Nacional de Trasplantes, Madrid, Spain; 4 Organ and Tissue Donation and Transplantation, NHS Blood and Transplant, Bristol, United Kingdom; 5 Gift of Life Donor Program, Philadelphia, PA, United States; 6 Department of Policy and Research, Dutch Transplant Foundation, Leiden, Netherlands; 7 Department of Anesthesia and Critical Care, AP-HP, Hôpital Lariboisière, Paris, France; 8 Université Paris Cité, INSERM U942 MASCOT, Paris, France; 9 Vall d’Hebron Research Institute, Barcelona, Spain; 10 Hospital Universitari Vall d’Hebron, Barcelona, Spain; 11 Simmons Transplant Institute, Baylor University Medical Center, Dallas, TX, United States; 12 Australian Centre for Health Law Research, Faculty of Business & Law, Queensland University of Technology, Brisbane, QLD, Australia; 13 Department of Anesthesia and Critical Care, AOU Città della Salute e della Scienza di Torino, Turin, Italy; 14 Division of Transplantation Surgery, CLINTEC, Karolinska Institutet, Stockholm, Sweden; 15 Unità di Chirurgia Epatobiliare e Trapianto Epatico, Università degli Studi di Padova, Padova, Italy; 16 School of Medicine, Deakin University, Geelong, VIC, Australia

**Keywords:** consensus, DCD (donation after circulatory death), deceased donation, donation, transplantation

There was a mistake in Table 1 as published. Incorrect percentages are included in two sections of the table. In addition, throughout the table, the percentages are shown to two decimal places, whereas they should be to a single decimal place. The corrected Table 1 appears below.

**TABLE 1:** Expert panel demographics.

Panelist characteristics	First-wave	Second-wave
​	n = 37	n = 35
Age		
18–30 years	0.0%	0.0%
31–40 years	10.8%	11.4%
41–50 years	24.3%	22.9%
51–60 years	35.1%	37.1%
>60 years	29.7%	28.6%
Gender		
Male	64.9%	62.9%
Female	35.1%	37.1%
Country of employment		
Argentina	5.4%	5.7%
Australia	10.8%	11.4%
Canada	5.4%	5.7%
China	5.4%	2.9%
France	8.1%	8.6%
Italy	10.8%	11.4%
Japan	2.7%	0.0%
Netherlands	8.1%	8.6%
New Zealand	2.7%	2.9%
South Africa	2.7%	2.9%
Spain	8.1%	8.6%
Sweden	2.7%	2.9%
United Kingdom	10.8%	11.4%
United States	13.5%	14.3%
Uruguay	2.7%	2.9%
Primary specialty		
Intensivist/critical care physician	40.0%	39.6%
Transplant or donation coordinator	18.0%	18.8%
Transplant surgeon	14.0%	12.5%
Anaesthesiologist	6.0%	6.3%
Ethicist or legal scholar	6.0%	6.3%
Others*	16.0%	16.7%
Primary patient population		
Adult medicine	67.6%	65.7%
Both paediatric and adult medicine	16.2%	17.1%
Not applicable to my role	16.2%	17.1%
Paediatrics	0.0%	0.0%
Primary sector of clinical practice for previous 12 months		
Public healthcare	73.0%	71.4%
Private healthcare	0.0%	0.0%
Both	16.2%	17.1%
Not Applicable	10.8%	11.4%
Type of hospital in which work		
Academic hospital	59.5%	60.0%
Non-academic hospital	8.1%	5.7%
Other academic centres and institutions	16.2%	17.1%
Other	16.2%	17.1%
Duration of experience with deceased donation or transplantation		
≥10 years	86.5%	85.7%
5–9 years	10.8%	11.4%
<5 years	2.7%	2.9%
Author contributions to academic publications in deceased organ donation or transplantation		
Yes	94.6%	94.3%
No	2.7%	2.9%
Not applicable	2.7%	2.9%
Participation as a principal investigator in a clinical trial relating to deceased organ donation or transplantation		
No	59.5%	62.9%
Yes	37.8%	34.3%
Not applicable to my role	2.7%	2.9%

* Values may not add up to 100% due to rounding errors.

The original version of this article has been updated.

